# Microtubule dynamics and post-translational modifications in cardiovascular diseases: mechanisms and therapeutic implications

**DOI:** 10.3389/fcvm.2026.1792687

**Published:** 2026-03-25

**Authors:** Lin Cui, Lulu Guo, Lufei Yang, Yuehao Zhang, Chunfen Ma

**Affiliations:** 1 Central Laboratory, The First Affiliated Hospital, Henan University of Chinese Medicine, Zhengzhou, Henan, China; 2 Clinical Medicine of Integrated Traditional Chinese and Western Medicine, Henan University of Chinese Medicine, Zhengzhou, China; 3 Traditional Chinese Medicine Clinic, The Third Affiliated Hospital of Henan University of Chinese Medicine, Zhengzhou, China

**Keywords:** atherosclerosis, cardiovascular disease, cytoskeleton, heart failure, microtubules, myocardial ischemia, tubulin

## Abstract

Microtubules, assembled from tubulin subunits, as a core component of the cytoskeleton, are extensively involved in fundamental biological activities such as cell shape maintenance, material transport, and cell division. Recent studies have revealed that abnormal microtubules dynamics and post-translational modifications (PTMs) of tubulin in cardiovascular diseases profoundly impact the function of cardiomyocytes and vascular smooth muscle cells, positioning the microtubule cytoskeleton as a key factor in cardiovascular pathophysiological processes. Specifically, alterations in microtubule stability and tubulin modifications influence cellular damage repair following myocardial ischemia but also regulate the development mechanisms of heart failure and atherosclerosis, demonstrating their multifaceted role in disease development. This systematic review examines microtubule dynamics and tubulin PTMs across various cardiovascular diseases and their underlying molecular mechanisms, with a focus on their potential as a therapeutic target. It aims to provide novel theoretical support and innovative insights for the diagnosis and treatment of cardiovascular diseases, thereby advancing clinical translation in this field.

## 1 Introduction

As the fundamental building block of microtubules, tubulin is indispensable for maintaining cellular morphology, facilitating intracellular transport, and mediating signal transduction. In the cardiovascular system, the dynamic equilibrium of the microtubule network is crucial for cellular homeostasis, enabling cardiomyocytes and vascular cells to adapt their structure and function in response to physiological and pathological stimuli. Cardiovascular diseases (CVDs), a leading cause of global mortality, involve multifaceted alterations in cellular architecture and function. Emerging evidence underscores that dysregulation of tubulin expression and remodeling of the microtubule cytoskeleton are pivotal in the pathogenesis of various CVDs, highlighting the microtubule system as a critical player in cardiovascular pathophysiology.

Vascular remodeling, a hallmark of progressive CVD, entails coordinated changes in vessel wall structure and composition, driven by hemodynamic forces and risk factors. This process involves dynamic behaviors of endothelial cells, fibroblasts, and vascular smooth muscle cells (VSMCs), alongside immune cell infiltration and inflammatory signaling. Microtubules, by regulating cell migration, proliferation, apoptosis, and signal transduction, directly influence these cellular activities. Key pathways such as Rho/ROCK, MAPK, and TGF-*β*/Smad interact with microtubule dynamics, suggesting that targeting microtubule regulation could offer novel strategies to modulate vascular remodeling (1). Cardiovascular aging is a major risk factor for CVD, characterized by structural and functional decline in the heart and vasculature, including myocardial hypertrophy, fibrosis, and metabolic shifts linked to microtubule remodeling and PTMs like acetylation and detyrosination, which mediate stress responses and contribute to heart failure, ischemia, and atherosclerosis (2–5). Furthermore, proteins such as the tubulin polymerization-promoting protein (Topp) regulate microtubule elongation and acetylation; its deficiency leads to microtubule dysfunction and exacerbates cardiovascular pathology by triggering inflammation (6).

Specific tubulin PTMs are intimately linked to cardiac and vascular dysfunction. In cardiomyocytes, increased microtubule detyrosination impairs contractile function and contributes to adverse remodeling post-myocardial infarction and in heart failure. Natural compounds like cinnamaldehyde can inhibit detyrosination and ameliorate cardiac hypertrophy, revealing the therapeutic value of targeting tubulin modifications (7, 8). Conversely, deficiency in tubulin-folding cofactor E (TBCE) induces endoplasmic reticulum stress in VSMCs, promoting vascular dysfunction and underscoring the importance of proper tubulin folding and microtubule assembly for vascular health (9).

In hemostasis and thrombosis, tubulin stability in platelets is a key regulatory node. The humanin analogue HNG inhibits platelet activation and thrombus formation by enhancing tubulin acetylation and stabilizing the microtubule cytoskeleton, pointing to the relevance of tubulin acetylation in thromboembolic disorders (10). Clinically, the anti-inflammatory drug colchicine, which inhibits tubulin polymerization, has been repurposed for the treatment of coronary artery disease, demonstrating the translational potential of microtubule-targeting agents in CVD management (11, 12).

**Figure 1:**
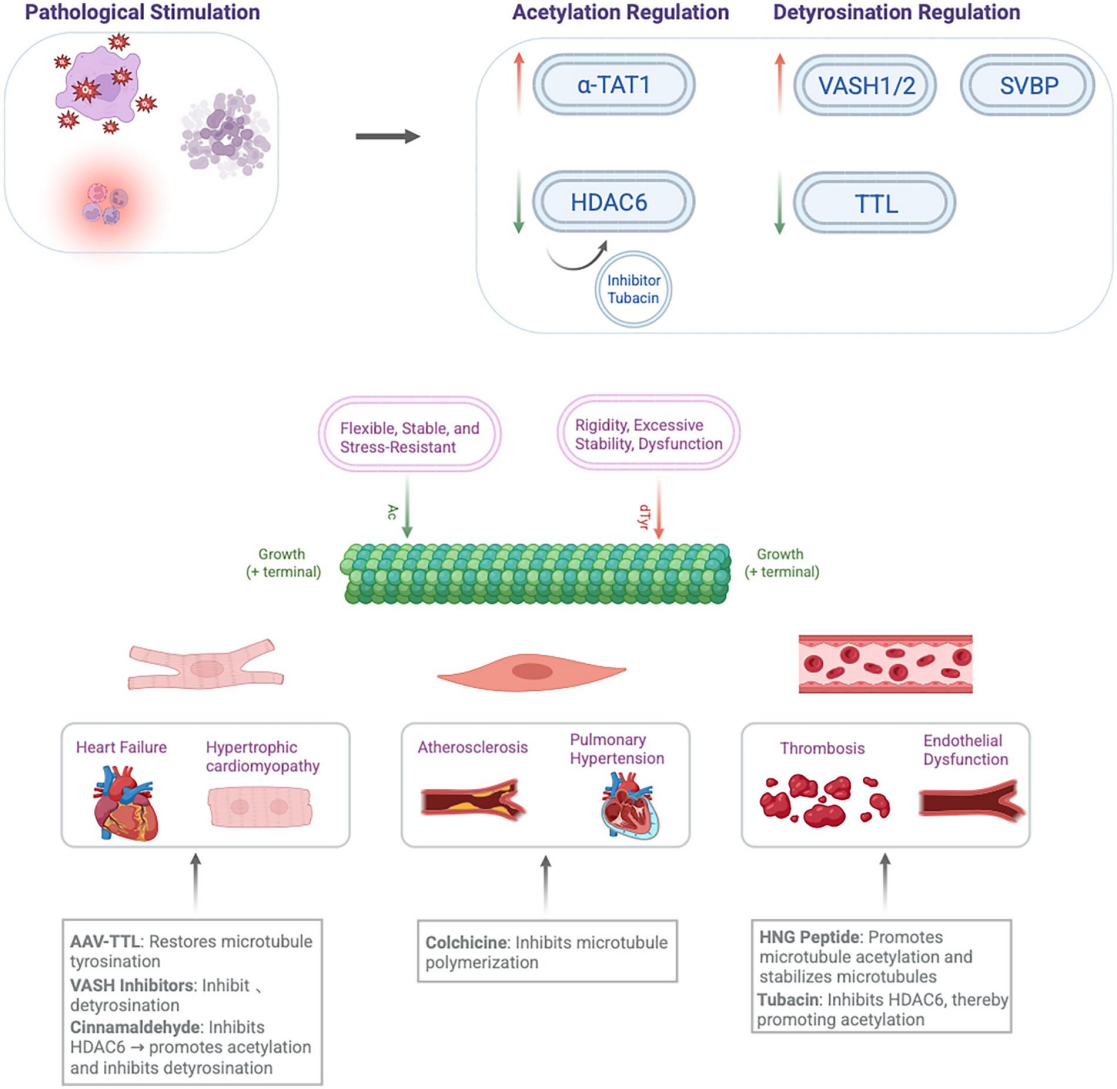
The central role of dynamic modulation of tubulin in cardiovascular diseases and targeted therapeutic strategies, including cinnamaldehyde for detyrosination inhibition, AAV-TTL for tyrosination restoration, and VASH inhibitors for improved contractility.

In summary, tubulin, through its expression levels, diverse PTMs, and dynamic reorganization of the microtubule network, plays a multifaceted role in the initiation and progression of CVDs. It integrates mechanical stability with signal transduction, stress sensing, and adaptive responses in cardiovascular cells. However, a systematic synthesis of how specific tubulin alterations drive distinct disease phenotypes, and a critical appraisal of the resulting therapeutic opportunities, are currently lacking. This review aims to (1): comprehensively summarize the changes in tubulin expression, PTMs, and microtubule dynamics across major CVDs, including myocardial ischemia, heart failure, and atherosclerosis (2); elucidate the consequent impact on cardiomyocyte and vascular cell function; and (3) evaluate the potential and challenges of therapeutic strategies targeting the microtubule cytoskeleton. By providing a unified mechanistic framework, we seek to highlight novel diagnostic and therapeutic avenues in cardiovascular medicine. A schematic overview of the central mechanisms discussed in this review is presented in Figure 1.

## 2 The tubulin protein in cardiovascular biology

### 2.1 Biological functions of tubulin in cardiovascular cells

While this section initially emphasizes cardiomyocytes and vascular smooth muscle cells (VSMCs) due to their primary roles in cardiac contraction and vascular tone regulation, subsequent subsections address microtubules in other key cardiovascular cell types, including endothelial cells, fibroblasts, and macrophages, to provide a comprehensive view.

#### 2.1.1 Structure of tubulin and microtubule dynamics

Microtubules are cylindrical polymers assembled from *α*/*β*-tubulin heterodimers, which serve as their fundamental structural units, organized from microtubule-organizing centers (MTOCs) such as the nuclear envelope and Golgi in cardiomyocytes [DOI: 10.1093/eurheartj/ehad205]. This organization facilitates perinuclear microtubule networks essential for mechanical support and transport [DOI: 10.1161/CIRCRESAHA.123.323174, DOI: 10.1038/s41467-022-35639-x]. A defining feature of microtubules is their dynamic instability—the stochastic growth and shrinkage at their plus ends. This dynamic behavior is essential for cellular processes such as morphogenesis, intracellular transport, and cell division. The transition from a curved conformation of free tubulin dimers to a straight protofilament during polymerization, coupled with GTP hydrolysis, underpins this dynamic property and governs microtubule stability (13–15). Beyond the core structure, the functional diversity of microtubules is expanded by a “tubulin code,” comprising various tubulin isotypes and a repertoire of post-translational modifications (PTMs), such as acetylation and detyrosination (16). These PTMs fine-tune microtubule mechanical properties and interactions with associated proteins. In cardiovascular cells, the acetylation of microtubules, for instance, enhances their flexibility and resilience against mechanical stress, aiding in the preservation of cellular integrity under load (5). Thus, the precise regulation of tubulin dynamics and its PTMs forms the foundation for normal cardiovascular cell function, and its dysregulation is intricately linked to disease pathogenesis (2).

#### 2.1.2 Role of tubulin in cardiomyocytes

In cardiomyocytes, the microtubule network provides crucial structural support, maintaining cellular shape and mechanical stability necessary for effective contraction and relaxation. The state of microtubule stabilization, heavily influenced by PTMs, directly impacts myocardial mechanics and calcium handling. Notably, elevated levels of microtubule detyrosination lead to an overly stabilized and densified network, increasing cardiomyocyte stiffness and thereby impairing primarily diastolic, and potentially systolic, function as supported by studies on tau aggregation and tyrosination disruption (17–19) [DOI: 10.1093/eurheartj/ehad205]. Furthermore, microtubules coordinate essential metabolic functions by regulating the positioning and function of mitochondria through interactions with motor proteins and other cytoskeletal elements, thus influencing ATP production and cellular energetics (20, 21). Tubulin acetylation serves a protective role by enhancing the cardiomyocyte's adaptability to mechanical and oxidative stress, promoting survival (5). Microtubules also participate in signal transduction; detyrosinated microtubules can influence calcium signaling pathways and downstream transcription factors, contributing to the development of pathological hypertrophy and heart failure (7, 22). Other modifications, such as nitrosylation of *β*2-tubulin, can promote microtubule disassembly and modulate contractile rhythms (23). Collectively, these findings position tubulin not merely as a static scaffold but as a dynamic regulator of myocardial metabolism, signaling, and stress adaptation, with its dysregulation being a central feature of various cardiac pathologies.

#### 2.1.3 Role of tubulin in vascular smooth muscle cells

Vascular smooth muscle cells (VSMCs) are central to vascular tone and structure. Tubulin, by forming the microtubule cytoskeleton, critically regulates VSMC behaviors including proliferation, migration, and phenotypic switching—key events in vascular remodeling. The acetylation and detyrosination states of tubulin influence VSMC stiffness and motility, driving the transition from a contractile to a synthetic phenotype, which is pivotal in atherosclerosis and vascular fibrosis (24, 25). Reorganization of the microtubule network also affects vascular tension and hemodynamics by modulating the interplay between the cytoskeleton and the extracellular matrix, thereby influencing VSMC contractility (26, 27). Microtubules act in concert with actin and intermediate filaments to integrate mechanical and biochemical signals. Moreover, tubulin-dependent processes regulate the synthesis and degradation of the extracellular matrix, impacting atherosclerotic plaque formation and stability (28, 29). Under pathological conditions, altered expression of microtubule-related proteins like KIF2C can disrupt calcium signaling and microtubule dynamics in VSMCs, promoting vascular calcification and stiffness (30). The microtubule network also modulates VSMC responses to inflammation, oxidative stress, and apoptosis, underscoring its multifaceted role in vascular pathophysiology (31, 32). In summary, tubulin in VSMCs is integral to both maintaining structural homeostasis and actively directing cellular responses that shape vascular health and disease, while similar dynamics in other cell types are discussed below.

#### 2.1.4 Role of tubulin in other cardiovascular cell types

In endothelial cells, microtubules regulate barrier function, migration, and nitric oxide production, with alterations in acetylation contributing to dysfunction in atherosclerosis and hypertension [DOI: 10.1007/s00018-025-05828-0, DOI: https://doi.org/10.1038/s41598-019-51024-z, DOI:10.1097/CCM.0b013e31827c0d8c]. Fibroblasts rely on microtubules for extracellular matrix remodeling and migration during fibrosis; dysregulated microtubule dynamics exacerbate cardiac remodeling in heart failure [DOI: 10.1073/pnas.0608030104, DOI:10.1083/jcb.201006009, DOI:10.3389/fcvm.2021.689101]. Macrophages use microtubules for polarization, phagocytosis, and cytokine release, where increased detyrosination promotes pro-inflammatory phenotypes in plaques [DOI: 10.3390/ijms24021373, DOI:10.3724/abbs.2024075]. These roles highlight the microtubule network's broad influence across cardiovascular tissues, with therapeutic implications for targeting PTMs in multi-cellular CVD contexts.

### 2.2 Alterations in tubulin expression and structure in cardiovascular diseases

#### 2.2.1 Changes in tubulin during myocardial ischemia and reperfusion injury

Myocardial ischemia and subsequent reperfusion injury represent critical pathological processes in cardiovascular disease, during which the remodeling of the cardiomyocyte cytoskeleton, particularly involving tubulin and the microtubule network, plays a pivotal role. During the ischemic phase, tubulin expression is downregulated in cardiomyocytes, leading to microtubule disassembly and disruption of the cytoskeletal architecture. This structural compromise impairs cellular mechanical stability and compromises signal transduction. Specifically, ischemia-induced oxidative stress and metabolic disturbance promote tubulin deacetylation and other modifications, which reduce microtubule stability and compromise cell survival (5).

The reperfusion phase is characterized by tubulin reorganization. The dynamic reconfiguration of the microtubule network critically influences cellular survival and apoptotic pathways. Altered microtubule stability during this phase not only affects the mechanical properties of cardiomyocytes but also modulates intracellular calcium signaling, thereby impacting the recovery of contractile function. Key post-translational modifications (PTMs), such as acetylation and deacetylation, serve as regulatory nodes in ischemia/reperfusion injury. Enhanced acetylation, for instance, can increase microtubule flexibility and antioxidant capacity, mitigate cytoskeletal damage and protect cardiomyocytes from excessive stress (5, 10).

The therapeutic potential of modulating these PTMs is highlighted by studies on natural compounds and synthetic peptides. Trans-cinnamaldehyde, for example, attenuates cardiac hypertrophy and reperfusion injury by inhibiting tubulin deacetylation, potentially through suppressing HDAC6 activity (7). Conversely, the humanin analogue HNG protects against injury by enhancing tubulin acetylation, stabilizing the microtubule structure, and inhibiting platelet activation and thrombosis (10). Collectively, the dynamic alterations in tubulin expression, network architecture, and PTM regulation during ischemia/reperfusion injury underscore its role as a promising therapeutic target.

In summary, myocardial ischemia and reperfusion injury are accompanied by a characteristic process of microtubule dynamic imbalance: from downregulation and depolymerization during ischemia to modification-dependent reorganization during reperfusion. Enhanced microtubule acetylation has been widely demonstrated as a protective strategy, with its core mechanisms involving cytoskeletal stabilization, improved calcium cycling, and inhibition of cell death. However, discrepancies persist across studies regarding the precise timing and extent of modifications such as microtubule deacetylation. These variations may stem from differences in model systems (e.g., whole heart vs. isolated cells), ischemia duration, and detection methods. Such inconsistencies suggest that future research requires more precise temporal control to map the complete kinetic profile of microtubule modifications.

#### 2.2.2 Tubulin abnormalities in heart failure

Heart failure is associated with well-documented abnormalities in tubulin expression and profound disorganization of the microtubule network in cardiomyocytes. A hallmark of failing myocardium is the increased abundance of tubulin, likely due to altered autoregulation, coupled with excessive microtubule stabilization [https://doi.org/10.3389/fcell.2022.837486]. This is particularly driven by a marked increase in the detyrosination of *α*-tubulin, resulting in a densified microtubule network. This pathological stabilization significantly increases the passive stiffness of cardiomyocytes, directly impairing both systolic and diastolic function (33, 34).

The shift towards a hyper-stable microtubule state has profound consequences for cardiomyocyte biomechanics and calcium handling. Excessively detyrosinated microtubules become more rigid, restricting cellular deformation during contraction and increasing myocardial stiffness, thereby exacerbating the heart failure phenotype (19). Inhibition of vasohibin (VASH) reduces detyrosination and improves relaxation in HFpEF models [DOI: 10.1126/scitranslmed.adm8842]. The activity of tubulin-modifying enzymes is central to this process. While histone deacetylase 6 (HDAC6) promotes deacetylation and can influence stability, tubulin tyrosine ligase (TTL) catalyzes the re-tyrosination of tubulin. Activation of TTL can reduce detyrosination, decrease cellular rigidity, and improve diastolic function, highlighting its potential as a therapeutic node (17, 18).

Furthermore, alterations in tubulin-associated proteins contribute to myocardial remodeling and fibrosis. For instance, dysregulated expression of tubulin-folding cofactor E (TBCE) promotes endoplasmic reticulum stress and VSMC proliferation, thereby participating in adverse myocardial remodeling (9). Following myocardial infarction, upregulated expression of microtubule affinity-regulating kinase 4 (MARK4) promotes tubulin detyrosination, enhances microtubule stabilization, and contributes to contractile dysfunction (35). These findings illustrate the multi-faceted regulatory role of the tubulin network in the pathophysiology of heart failure.

Existing evidence strongly indicates that “microtubule disease”—characterized by excessive stabilization and densification of the microtubule network driven by *α*-tubulin overexpression and de-tyrosine modification—is prevalent in failing hearts. This represents a key structural basis for increased myocardial cell rigidity and impaired diastolic function. Targeting modifying enzymes (e.g., activating TTL or inhibiting HDAC6/MARK4) to restore microtubule dynamic equilibrium has emerged as a widely recognized therapeutic approach. Preclinical studies have shown that downregulating MARK4 expression promotes mitochondrial biogenesis and reduces myocardial damage in rat models of infarction [DOI: 10.1155/2023/5677597], though specific small-molecule inhibitors are not yet clinically available. The primary current debate and knowledge gap lies in whether the initiation mechanisms and core modification types of microtubule abnormalities exhibit specificity across different etiologies of heart failure (e.g., stress overload, ischemic, diabetic), which directly impacts the development of precision treatment strategies.

#### 2.2.3 Tubulin regulation in atherosclerosis and vascular pathologies

Tubulin expression and regulation are also critically involved in the pathogenesis of atherosclerosis and related vascular diseases. In vascular smooth muscle cells (VSMCs), upregulation of tubulin expression promotes cell migration and proliferation, which are key events in the formation and progression of atherosclerotic plaques (9, 24).

Remodeling of the microtubule network contributes to vascular endothelial dysfunction and inflammation. The acetylation status of tubulin influences endothelial cell function, partly through modulating endothelial nitric oxide synthase (eNOS) expression, thereby affecting endothelium-dependent vasodilation (36). Deficiency in TBCE induces endoplasmic reticulum stress in VSMCs, driving excessive proliferation and vascular wall thickening, culminating in vascular dysfunction (9).

Tubulin-mediated cytoskeletal changes further influence plaque formation and stability. PTMs like detyrosination and acetylation affect cellular migration and inflammatory responses within the plaque, regulating the activity of resident cells (11, 12). The clinical efficacy of colchicine, an anti-inflammatory drug that inhibits tubulin polymerization, in reducing atherosclerotic cardiovascular events underscores the therapeutic validity of targeting microtubule dynamics in atherosclerosis (11, 12).

Additionally, tubulin dynamics are closely linked to the inflammatory response in vascular endothelial cells. Tubulin polymerization facilitates the interaction between endothelial cells and immune cells, amplifying inflammatory signaling and potentially promoting plaque instability (32, 37). Tubulin-related proteins, such as TPPP3, exacerbate endothelial oxidative damage and inflammation by modulating the mitochondrial channel VDAC1, highlighting the role of the tubulin network in vascular inflammation (31). In summary, upregulated tubulin expression and subsequent microtubule network remodeling drive VSMC dysregulation, endothelial dysfunction, and inflammation, positioning tubulin as a significant target for anti-atherosclerotic therapy.

In atherosclerosis, microtubules exhibit a dual role dependent on cell type: within VSMCs, microtubule remodeling drives pathological proliferation, migration, and phenotypic switching, whereas in endothelial cells, microtubule dysfunction exacerbates inflammatory responses and oxidative stress. Inhibiting microtubule polymerization with drugs such as colchicine to suppress inflammation and proliferation has gained consistent support from both preclinical and clinical studies. Current controversies primarily center on the early disease stages, where inconsistent reports exist regarding the expression and functional changes of microtubule-associated proteins (e.g., TBCE, TPPP3). This may reflect the heterogeneity across different plaque development phases or vascular beds, representing a key focus for future research clarification.

In summary, tubulin exhibits distinct expression and modification patterns across various cardiovascular diseases, all of which disrupt microtubule dynamics equilibrium and ultimately lead to cellular dysfunction (Tables 1, 2).

**Table 1:** Alterations in tubulin expression, post-translational modifications, and microtubule dynamics in major cardiovascular diseases.

Disease/Pathological Process	Primary Cell Types Involved	Key Alterations in Tubulin/Microtubules	Functional Consequences & Structural Remodeling	Associated Signaling Molecules/Pathways	Potential Therapeutic Strategies/Compounds (Key Refs.)
Myocardial Ischemia/Reperfusion Injury	Cardiomyocytes	• Downregulation of Tubulin expression • Decreased acetylation • Increased detyrosination (proposed)	• Disassembly of the microtubule network and loss of cytoskeletal integrity • Reduced mechanical stability and impaired calcium signaling • Promotion of cellular apoptosis	• Elevated HDAC6 activity • Oxidative stress and metabolic disturbance	• Trans-cinnamaldehyde: Inhibits HDAC6, enhances acetylation, and attenuates injury (7) • HNG peptide: Promotes microtubule stabilization via acetylation (10)
Heart Failure (esp. HFpEF)	Cardiomyocytes	• Overexpression of *α*-tubulin • Markedly enhanced detyrosination • Hyper-stabilization of microtubules	• Over-stabilized and densified microtubule network • Increased passive stiffness of cardiomyocytes, impairing diastolic function • Disrupted mitochondrial trafficking and energy metabolism	• Reduced activity of Tubulin tyrosine ligase (TTL) • Upregulation of MARK4 • Altered activity of vasohibin/SVBP complex	• TTL activation: Restores tyrosination, improves diastolic compliance (17, 18) • MARK4 inhibition: Attenuates pathological detyrosination (preclinical) (35) • VASH inhibition: Reduces detyrosination, enhances relaxation [DOI: 10.1126/scitranslmed.adm8842]
Atherosclerosis & Vascular Remodeling	Vascular Smooth Muscle Cells (VSMCs)	• Upregulation of Tubulin expression • Altered acetylation/detyrosination balance, driving phenotypic switching	• Microtubule remodeling promotes transition from contractile to synthetic phenotype • Enhanced migration, proliferation, and extracellular matrix production • Participation in inflammatory responses	• TBCE deficiency-induced ER stress • Activation of Rho/ROCK, TGF-*β*/Smad pathways	• Colchicine: Inhibits microtubule polymerization, exerts anti-inflammatory and anti-proliferative effects (11, 12)
Cardiovascular Aging	Endothelial cells, VSMCs, Cardiomyocytes	• Microtubule network remodeling • Loss of balance in Tubulin PTMs (e.g., acetylation)	• Reduced cytoskeletal adaptability, contributing to endothelial dysfunction and decreased vascular compliance • Cardiomyocyte hypertrophy, fibrosis, and diminished resistance to mechanical/oxidative stress	• Age-associated oxidative stress and mechano-signaling pathways	• HDAC6 modulation: Restoring microtubule dynamics represents a potential strategy (2, 3)
Thrombosis	Platelets	• Reduced acetylation, leading to microtubule destabilization	• Hyper-activation of platelets, increased aggregation and adhesion, promoting thrombus formation	• Dysregulated microtubule-motor protein interactions	• HNG peptide: Stabilizes platelet microtubules via enhanced acetylation, inhibiting activation (10)

**Table 2:** Comparative analysis of microtubule-targeted therapies in preclinical CVD models.

Therapy	Preclinical Model	Key Outcomes
Colchicine (MT polymerization inhibitor)	Mouse models of myocardial infarction (MI) and atherosclerosis; rat models of viral myocarditis	Reduced inflammation, improved LV function, decreased plaque progression; anti-inflammatory effects via NLRP3 inflammasome inhibition
Paclitaxel (MT stabilizer)	Rodent models of heart failure (HF) and ischemia-reperfusion (I/R) injury	Suppressed excessive MT depolymerization, reduced cardiomyocyte apoptosis and inflammation, improved cardiac function
Trans-cinnamaldehyde (detyrosination inhibitor via HDAC6)	Mouse models of cardiac hypertrophy and I/R injury	Attenuated hypertrophy, reduced detyrosination, improved structure and function
HNG peptide (acetylator, MT stabilizer)	Rodent models of I/R injury and thrombosis	Enhanced MT acetylation, stabilized structure, inhibited platelet activation, reduced thrombus formation
AAV-TTL (tyrosination activator)	Mouse models of hypertrophic cardiomyopathy (HCM) and HF	Restored tyrosination, reduced stiffness, improved diastolic function and contractility
VASH inhibitors (detyrosination inhibitors)	Rat models of HF with preserved ejection fraction (HFpEF)	Reduced detyrosination, improved myocardial relaxation and heart function
Tubacin (HDAC6 inhibitor, acetylator)	Mouse models of diabetic endothelial dysfunction	Enhanced MT acetylation, upregulated eNOS, improved endothelial function, attenuated vascular injury
MARK4 inhibitors (e.g., lisinopril or genetic knockdown)	Rat models of post-MI HF	Reduced detyrosination, promoted mitochondrial biogenesis, improved contractility
Parthenolide (detyrosination suppressor)	Preclinical HF models	Suppressed detyrosinated MTs, reversed cardiomyocyte damage, improved function
Epothilone-Y (detyrosination inhibitor)	Mouse models of DCM and HCM	Reduced nuclear invagination, rescued sarcomere-nuclear strain, decreased YAP1 translocation

### 2.3 Advances in targeting tubulin for cardiovascular therapy

**Figure 2:**
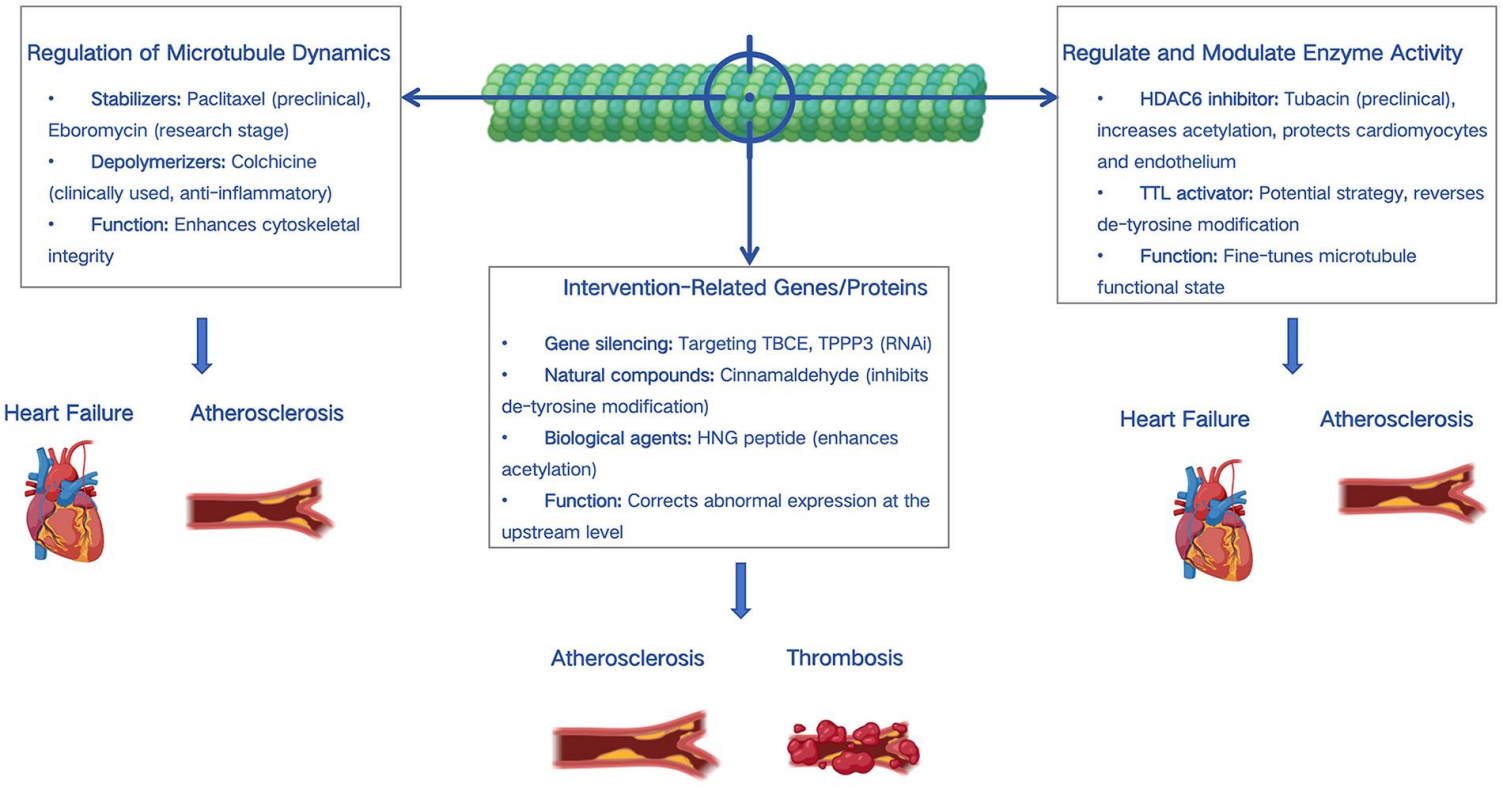
Therapeutic strategies targeting the microtubule cytoskeleton.

Various therapeutic strategies targeting the aforementioned tubulin alterations are under investigation, as summarized in Figure 2.

#### 2.3.1 Potential applications of microtubule stabilizing agents in cardiovascular disease

Microtubule stabilizing agents, which modulate cytoskeletal integrity, have shown promising potential in cardiovascular therapeutic research. Classical stabilizers such as paclitaxel enhance microtubule stability, thereby improving the structural integrity and functional performance of cardiomyocytes. In models of cardiovascular disease, paclitaxel and related stabilizers can suppress excessive microtubule depolymerization, reduce cardiomyocyte apoptosis and inflammation, and ultimately improve cardiac function. For example, studies demonstrate that microtubule stabilization effectively regulates the transmission of mechanical load and intracellular signaling in cardiomyocytes, alleviating myocardial dysfunction caused by microtubule destabilization (2). Furthermore, certain natural compounds such as trans-cinnamaldehyde can inhibit microtubule detyrosination, attenuating cardiac hypertrophy and thereby improving cardiac structure and function (7). Microtubule stabilizers also help mitigate calcium overload in cardiomyocytes by modulating calcium channels and related signaling pathways, improving sarcomere shortening and overall cardiac pumping capacity.

However, the clinical translation of microtubule stabilizers faces challenges related to dosage optimization and side effects. High doses of drugs such as paclitaxel can induce toxicities like myelosuppression and neurotoxicity, limiting their long-term use. Therefore, current research focuses on optimizing dosages and developing stabilizers with higher selectivity and lower toxicity. Novel agents such as the HDAC6 inhibitor Tubacin, which selectively modulates microtubule acetylation, improve endothelial function and cardiomyocyte status with a more favorable safety profile, highlighting a promising therapeutic avenue (36). Additionally, combination strategies that pair microtubule stabilizers with other cardiovascular drugs are being explored to achieve synergistic effects while minimizing overall toxicity.

In summary, the protective role of microtubule stabilizers in cardiovascular disease has been substantiated by multiple experimental studies. By regulating microtubule structure and dynamic equilibrium, these agents suppress inflammation and apoptosis, thereby improving cardiac performance. Future efforts should prioritize dose optimization, enhanced target specificity, and the development of combination regimens to transform microtubule stabilizers into clinically safe and effective therapies for cardiovascular diseases (2, 7, 36).

#### 2.3.2 Tubulin-Modifying enzymes and their regulatory mechanisms

Post-translational modifications (PTMs) of tubulin, such as acetylation and deacetylation, play crucial regulatory roles in the pathogenesis of cardiovascular diseases. The expression and activity of tubulin-modifying enzymes—particularly acetyltransferases and deacetylases like HDAC6—are frequently dysregulated in cardiovascular disease models, impacting microtubule stability and cellular function. HDAC6 promotes microtubule deacetylation, reducing stability and contributing to dysfunction in both cardiomyocytes and vascular endothelial cells. Conversely, inhibition of HDAC6 with agents such as Tubacin enhances microtubule acetylation, upregulates endothelial nitric oxide synthase (eNOS) expression, and improves endothelial function, thereby attenuating vascular injury in diabetic models (36). Moreover, the acetylation state of tubulin influences cardiomyocyte adaptation to mechanical and oxidative stress; increased acetylation enhances microtubule flexibility, preserves cytoskeletal integrity, and helps maintain cellular homeostasis (5).

Enzymes that regulate tubulin PTMs represent novel and promising therapeutic targets for cardiovascular disease. The development of small-molecule inhibitors and activators is advancing rapidly. Selective HDAC6 inhibitors, for instance, not only enhance microtubule stability but also modulate downstream signaling pathways, suppressing cardiomyocyte apoptosis and inflammation. The humanin analogue HNG further exemplifies this approach, as it enhances platelet microtubule acetylation, inhibits platelet activation, and reduces thrombus formation, underscoring the importance of tubulin-modifying enzymes in antithrombotic and cardiovascular protection (10).

Furthermore, the expression and activity of these enzymes are themselves modulated by cardiovascular pathological states. Altered tubulin PTMs and corresponding enzyme expression have been observed in models of heart failure, ischemia-reperfusion injury, and pulmonary hypertension (8, 38). These findings provide a strong rationale for targeting tubulin-modifying enzymes in clinical translation. Future research should focus on elucidating the regulatory networks of these enzymes, optimizing the selectivity and safety of small-molecule modulators, and exploring synergistic effects with existing therapies to enable precise modulation of tubulin PTMs and improve cardiovascular outcomes (5, 10, 36).

#### 2.3.3 Gene regulation and tubulin-related therapeutic strategies

Dysregulation of tubulin family gene expression has been implicated in various cardiovascular diseases, contributing to pathological processes such as myocardial hypertrophy, heart failure, and vascular dysfunction. Genome-wide association studies have revealed that polymorphisms in the gene encoding tubulin-folding cofactor E (TBCE) are inversely correlated with endothelial function. TBCE deficiency induces endoplasmic reticulum stress in vascular smooth muscle cells, leading to vascular dysfunction and arterial wall thickening, highlighting the critical role of TBCE and tubulin homeostasis in vascular disease (9). Additionally, tubulin-associated proteins such as TPPP3 exacerbate endothelial oxidative stress and apoptosis by modulating VDAC1 stability, further implicating tubulin-related genes and their regulatory networks as potential therapeutic targets (31).

RNA interference (RNAi) and gene-editing technologies offer advanced tools for modulating tubulin expression. RNAi can be used to specifically silence disease-associated tubulin genes, ameliorating pathological changes in cardiomyocytes and vascular cells. Gene-editing techniques like CRISPR/Cas9 allow for more precise genetic modifications to correct aberrant expression and restore cellular function. Although still in exploratory stages for cardiovascular applications, these gene-regulatory strategies have shown considerable promise in preclinical studies, particularly in modulating the expression of microtubule-associated proteins, modifying enzyme activity, and fine-tuning cellular responses (9, 31).

Combination therapeutic strategies are also gaining attention. Integrating gene regulation with pharmacological interventions—such as microtubule stabilizers or modifiers of PTM enzymes—could enable multi-targeted, synergistic effects, enhance efficacy while reduce the side effects associated with monotherapies. Furthermore, personalized medicine approaches that incorporate genomic and phenotypic data could facilitate the design of tailored tubulin-targeted regimens, improving both safety and therapeutic outcomes. Future research should aim to systematically decipher the gene regulatory mechanisms of tubulin, optimize gene-targeting tools for cardiovascular applications, and foster interdisciplinary collaborations to accelerate the clinical translation of tubulin-related therapeutic strategies (9, 31).

## 3 Conclusions and translational outlook

The collective evidence presented in this review firmly establishes tubulin and the microtubule cytoskeleton as critical determinants of cardiovascular cellular homeostasis, whose dysregulation is integral to the pathogenesis of major diseases such as myocardial ischemia, heart failure, and atherosclerosis. Far from being a passive structural element, tubulin functions as a dynamic signaling hub, with its expression levels, polymerization dynamics, and intricate post-translational modification “code” directly influencing mechanical stability, organelle function, and cellular adaptation to stress. This multifaceted role not only deepens our molecular understanding of cardiovascular pathophysiology but also robustly validates the microtubule system as a promising and druggable therapeutic target.

A synthesis of current literature reveals several core, convergent findings, including microtubule detyrosination as a key druggable target through TTL delivery (e.g., AAV-TTL) or VASH inhibition to restore dynamic balance [DOI: 10.1126/scitranslmed.adm8842]. Across diverse disease models, an imbalance in microtubule network stability—whether pathological hyper-stabilization in cardiomyocytes or altered dynamics in vascular cells—emerges as a common structural node driving cellular dysfunction, necessitating careful temporal modulation in therapies. Importantly, microtubule stabilization offers short-term benefits by improving initial cardiomyocyte survival through decreased apoptosis, but long-term over-stabilization can impede contractility and exacerbate heart failure phenotypes. The pathological significance of specific tubulin modifications, particularly the protective role of acetylation and the deleterious effects of excessive detyrosination, has been consistently demonstrated. Furthermore, the therapeutic potential of modulating this system is strongly supported by preclinical evidence ranging from natural compounds to clinically used agents like colchicine.

However, the path to clinical translation is illuminated by these insights yet obscured by significant challenges and unresolved controversies. Key among these is the context-dependent nature of microtubule remodeling, where observed changes vary with disease stage, etiology, and experimental model, cautioning against oversimplified generalizations. Major knowledge gaps persist regarding the functional specificity of different tubulin isotypes in cardiovascular cells, the causal vs. consequential role of microtubule alterations in disease progression, and their precise crosstalk with other cytoskeletal networks and metabolic pathways.

To bridge the gap between compelling mechanistic insight and tangible clinical benefit, future research must adopt a more precise, integrative, and translational focus, including testing hypotheses on PTM specificity via advanced models like organoids and AI-driven predictions. Priorities include leveraging advanced technologies—such as super-resolution imaging *in vivo*, single-cell omics, and inducible genetic models—to achieve spatiotemporal precision in understanding microtubule behavior. Concurrently, translational efforts must innovate strategies to enhance the specificity and safety of interventions, including the development of tissue-targeted delivery systems for microtubule modulators and the exploration of synergistic combination therapies. The pursuit of biomarkers for microtubule dysfunction and the utilization of patient-derived cellular models will be crucial for personalizing these approaches and stratifying patient populations.

In conclusion, while tubulin has unequivocally emerged from the shadow of a static scaffold to occupy center stage as a dynamic regulator in cardiovascular disease, realizing its full therapeutic promise demands a concerted effort. This requires resolving existing controversies through sophisticated experimental design, fostering interdisciplinary collaboration, and maintaining an unwavering focus on overcoming the practical barriers to clinical application. By doing so, targeting the microtubule cytoskeleton holds the potential to deliver novel, mechanism-based strategies that could significantly alter the therapeutic landscape for cardiovascular diseases.
